# RETRACTION: “H‐Ras Oncogene Expression and Angiogenesis in Experimental Liver Cirrhosis”

**DOI:** 10.1155/grp/9871791

**Published:** 2026-04-01

**Authors:** Gastroenterology Research and Practice

RETRACTION: G. Özlem Elpek, B. Ünal, and S. Bozova, “H‐Ras Oncogene Expression and Angiogenesis in Experimental Liver Cirrhosis,” *Gastroenterology Research and Practice*, vol. 2013 (2013). https://doi.org/10.1155/2013/868916.

The above article, published online on 22 August 2013 in Wiley Online Library (wileyonlinelibrary.com), has been retracted following the agreement of the journal’s Chief Editor Michel Kahaleh; and John Wiley & Sons Ltd.

The retraction has been agreed following an investigation of the issues raised by *Penicillium citreoviride* on PubPeer [1], which highlighted concerns related to Figure 1 in the manuscript.

More specifically, the illustrations of Angiogenesis for groups I and II share multiple identical features, even though they represent different experimental conditions.

The authors were contacted to elaborate on these concerns and provide the raw data supporting the conclusions of the article; however they were unable to do so. As a result of this investigation, the results of the article are no longer considered reliable.

The authors disagree with this retraction.

## References

[1] Penicillium citreoviride , H-Ras Oncogene Expression and Angiogenesis in Experimental Liver Cirrhosis, PubPeer. (2021) https://pubpeer.com/publications/FBB5B079F7F06D3F983450215B20DD. 10.1155/2013/868916 PMC3819787 24235967

